# Accuracy and safety verification of ovarian reserve assessment technique for ovarian tissue transplantation using optical coherence tomography in mice ovary

**DOI:** 10.1038/srep43550

**Published:** 2017-03-08

**Authors:** Seido Takae, Kosuke Tsukada, Yorino Sato, Naoki Okamoto, Tai Kawahara, Nao Suzuki

**Affiliations:** 1 Department of Obstetrics and Gynecology, St. Marianna University School of Medicine, Kawasaki, Japan; 2 Graduate School of Fundamental Science and Technology, Keio University, Minato, Japan.

## Abstract

Except for histological study, there are currently no suitable techniques available for the detection and identification of primordial follicles in ovary of primary ovarian insufficiency patients who have undetectable AMH levels. Also, the ability to locate and quantify follicles on ovarian cortex strips, without fixation, is valuable for patients who could undergo subsequent successful ovarian tissue transplantation. Although optical coherence tomography (OCT) is a well-established high resolution imaging technique without fixation commonly applied in biomedicine, few reports are available on ovarian tissue imaging. In present study, we established standard OCT follicle images at each developmental stage, including the primordial follicle, and demonstrated the efficacy of OCT to estimate IVF outcome in transplanted mice ovary like ovarian reserve tests. Unfortunately, the current commercial OCT could not be used to accurate follicle count the number of follicles for whole ovary, because the maximum depth of examination was 100 μm. And we demonstrated the safety of OCT examination, it did not affect IVF outcome and birth defect rate, and reproductive ability. Although there is room for improvement, these findings will be first step to bring OCT examination a step closer to clinical application for measuring true ovarian reserve and localizing follicles.

Reproductive aging is related to both a quantitative and a qualitative reduction of the primordial follicle pool^1^. The quantitative ovarian reserve may vary substantially between women of the same chronological age^2^ because the initial endowment of primordial follicles, and rate of follicle loss, are highly variable between individuals^3^. Besides the age of patients, ovarian reserve tests (ORTs) have been developed to assess ovary function. These tests include basal FSH level, basal inhibin B, basal estradiol level, the clomiphene citrate challenge test (CCCT), the GnRH agonist challenge test (GAST), exogenous FSH ovarian reserve test (EFORT), the ovarian antral follicle count (AFC), and serum level of anti-Müllerian hormone (AMH)^4^,^5^. Several of these tests have become part of the standard pretreatment assessment for IVF (*in vitro* fertilization) designed to assess oocyte yield in response to gonadotropins and the probability of ongoing pregnancy after IVF^5^,^6^. In particular, there are strong indications that AMH and AFC may serve as good candidate markers for the determination of the ovarian reserve; for example, the area under the receiver operating curve (AUC) of AMH and AFC were 0.78 and 0.76 respectively^7^. However, AMH and AFC have limitations in assessing true ovarian reserve for immoderate diminished ovarian reserve patients, despite being reliable tests. In fact, around 100 to 1000 follicles remain in the ovary of patients who have undetectable serum AMH levels^4^. At this time, there are no tests that can confirm the true number of remaining follicles in the ovary except histological study with formaldehyde fixation. Indeed, knowing the true number of remaining follicles would be helpful for primary ovarian insufficiency patients (POI) in making decisions about continued fertility treatment. Furthermore, knowing the true number and localization of follicles in the ovarian tissue strips would be also helpful for patients who receive ovarian tissue transplantation as fertility treatment after cancer therapy or fertility treatment with *in vitro* activation (IVA)^8^,^9^, because they could receive an ovarian tissue transplantation with most suitable ovarian tissue containing the largest number of follicles.

Optical coherence tomography (OCT) is an emerging high-resolution and non-invasive imaging technique that has many applications in biomedicine^10^,^11^. The OCT technique measures back-scattered light from microstructural features within the examined tissues in the scale of several to tens of microns with a penetration depth of 1–3 mm^11^,^12^. Recently, OCT has been used to image tissues in the body for clinical examination including areas that can be accessed either directly, or via an endoscope or catheter, such as ophthalmology^13^,^14^, dentistry^15^,^16^, gastrointestinal tract^17^,^18^, coronary blood vessels^19^,^20^, colon^21^, breast^22^,^23^, and so forth^11^,^12^. In addition, some researchers have attempted OCT technique application in ovary investigation^10^,^12^,^24^,^25^,^26^,^27^.

Using the latest OCT equipment, our study aims to establish a protocol for standardized follicles imaging at each developmental stage, to assess the efficacy of OCT to estimate IVF outcome like ovarian reserve tests, and to investigate the safety of OCT for optimal gamete viability.

## Result

### Standard OCT images of mice ovary on individual age

**Figure 1:**
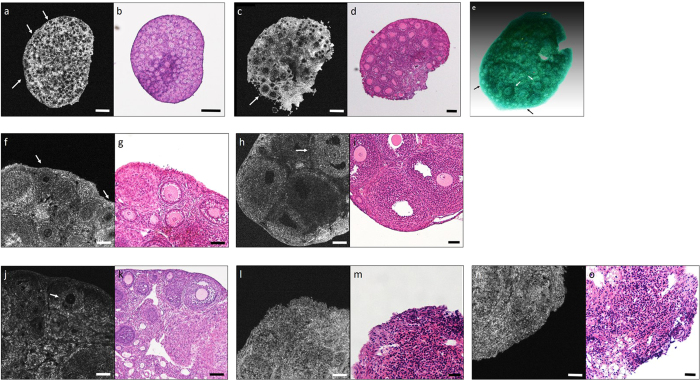
Optical coherence tomography (OCT) images and corresponding histology images at each ages of mice ovary. OCT images: (**a**,**c**,**f**,**h**,**j**,**l**,**n**), Histology: (**b**,**d**,**g**,**i**,**k**,**m**,**o**), 3D OCT image: (**e**) Histological images: many primordial follicles (white arrows) were seen in (**a**) day 3 mouse ovaries, primary to early secondary follicles (white arrows) were seen in day 10 mouse ovaries (**c**), secondary follicles and antral follicles (white arrows) were seen in (**f**,**h**) day 21 mouse ovaries. Image (**e**) is 3D imaging demonstrates the stereoscopic structure of (**c**). There are many primordial and primary follicles (black arrows) and early secondary follicles (white arrow). There were few secondary follicles (white arrows), however, seen in the OCT images of 30 weeks old mouse (**j**), and 50 weeks old mice (**l**,**n**) corresponding with histology (**k**,**m**,**o**, respectively). Each bar denotes 100 μm.

We could detect each developing follicles by OCT examination, including primordial follicles. And also, we established the standard OCT images of each developing stage follicles shown as Fig. 1. It shows a set of OCT images and H&E-stained histology images from healthy ICR mice at various ages. The OCT image from the day 3 mouse shows many primordial follicles consistent with the H&E-stained images (Fig. 1a,b). OCT images from the day 10 mice show primary and early secondary follicles consistent with the H&E-stained images (Fig. 1c,d). Also, 3D imaging of the day 10 mice is shown in Fig. 1e. The OCT images from the day 21 mice (Fig. 1f,g) show the late secondary and antral follicles consistent with the H&E-stained images (Fig. 1h,i). In 30 weeks old mice, some pre-antral follicles and corpus luteum are shown in the OCT images (Fig. 1j) and the H&E-stained images (Fig. 1k). In addition, in the 50 weeks old mice, a no follicles were visible in the OCT images (Fig. 1l,n) and the H&E-stained images (Fig. 1m,o).

### IVF for ovarian tissue transplantation outcome after OCT examination

**Figure 2:**
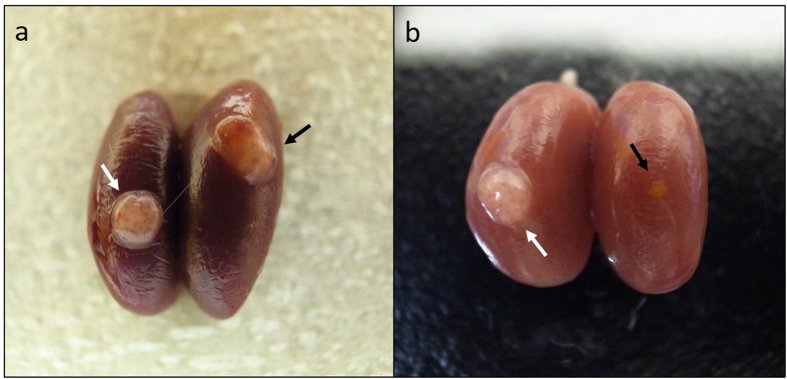
Ovaries transplanted into the kidney capsule 17 days after transplantation. (**a**) Day 3 ovary was transplanted into left side kidney after OCT examination. On the other hands, aged mice ovary (30 weeks) was transplanted into the right side kidney after OCT examination. (**b**) Day 3 ovary was transplanted into left side kidney after OCT examination. On the other hands, aged mice ovary (50 weeks) was transplanted into the right side kidney after OCT examination. In contrast, many developed follicles were seen on left side of the ovary (white arrows). Especially, aged mice ovary (50 weeks) was absorbed due to severe follicle loss (black arrow).

**Figure 3:**
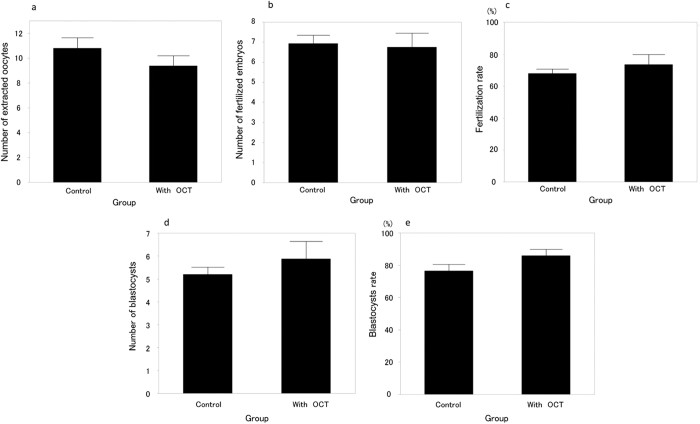
Comparison of IVF outcomes using transplanted ovaries with or without OCT examination. Without OCT examination of day 3 ovaries were defined as “control” group (n = 15). And with OCT examination of day 3 ovaries were defined as “with OCT” group (n = 10). Using statistical analysis, there were no significant differences between “control” and “with OCT” groups, in terms of number of extracted oocytes (**a**) (*p* = 0.29), fertilized embryos (**b**) (*p* = 0.81), blastocyst number (**d**) (*p* = 0.34), fertilization rate (**c**) (*p* = 0.35) or blastocyst rate (**e**) (*p* = 0.14). The level of significance was set at *p* < 0.05. In addition, there were no extracted oocytes from transplanted 30 and 50 weeks old mice ovaries (n = 5 each).

**Figure 4:**
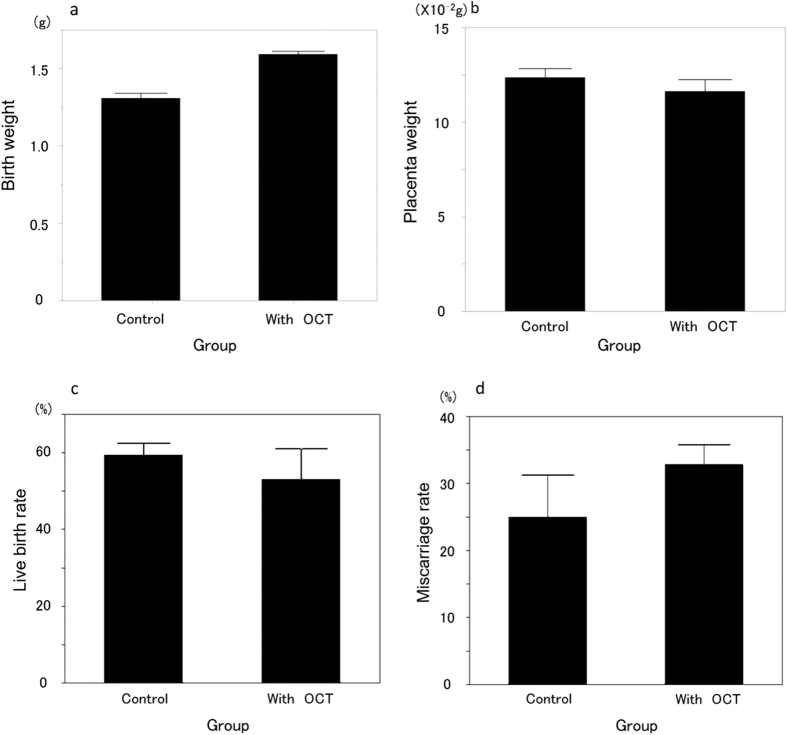
Comparison of newborn and placenta weight from transplanted ovaries with or without OCT examination, including live birth and abortion rate. Although there was a significant difference in the birth weight of newborn mice, there were no differences in the placenta weight, the live birth rate (*p* = 0.51), or the abortion rate (*p* = 0.41) among groups with or without OCT examination (n = 10, 15 respectively).

Figure 2a and b shows a transplanted ovary pair two weeks after transplantation following OCT examination. No oocytes could be retrieved from 30 or 50 weeks old mice ovaries (n = 5 each). Among the day 3 ovaries transplanted into kidney capsule with (n = 10)/without OCT examination (without OCT examination: control group, n = 15), there was no significant difference between the number of extracted oocytes from the transplanted ovaries (10.8 ± 0.8 and 9.4 ± 0.8, *p* = 0.29), the mean number of fertilized oocytes (6.9 ± 0.4 and 6.8 ± 0.7, *p* = 0.81), the fertilization rate (67.9 ± 2.7% and 73.5 ± 6.3%, *p* = 0.35), the number of blastocysts (5.2 ± 0.3 and 5.9 ± 0.8, *p* = 0.34), or the blastocyst rate (76.5 ± 4.1% and 87.3 ± 5.8%, *p* = 0.14) (Fig. 3a–e). Four 10 weeks old female mice received transplants of 48 blastocysts resulting in the Cesarean delivery of 27 normal mice. None of the delivered mice displayed gross deformities or abnormal placentas. The birth and placenta weight from transplanted ovaries with OCT were 1.59 ± 0.02 g and 0.11 ± 0.01 g, respectively. Compared with the control group (32 transplanted blastocysts among two host mice), there was a significant difference only in terms of birth weight (control group: birth weight 1.31 ± 0.03 g, placenta weight 0.12 ± 0.01 g) (Fig. 4a,b). Meanwhile, the delivery rate (control: 59.3 ± 3.1%, with OCT: 53.1 ± 7.9%) and the miscarriage rate (control: 25.0 ± 6.3%, with OCT: 32.9 ± 3.0%) were unchanged (Fig. 4c,d). All 5 mated female mice became pregnant and delivered healthy litters of 11.2 ± 0.4 progeny (female 5.4 ± 0.2 and male 5.8 ± 0.4).

## Discussion

Although it has been demonstrated that OCT could be used for ovarian tissue characterization, we found that traditional OCT equipment had insufficient resolution for detailed visualization of ovarian tissue characteristics such as primordial follicles^10^,^11^,^12^,^28^. Recently, researchers reported the efficacy of OCT to detect cancer metastasis and to confirm follicle density^27^. However, there is still little detailed information for standard imaging practice in examining ovarian tissue characteristics with OCT. Therefore, in present study we established more detailed images of ovaries including 3D images using OCT equipment on the mice ovaries. In addition, the present study is the first to report the primordial follicle detection on fresh (rather than fixed and embedded) ovary, which strongly indicates the viability of OCT examination for clinical application in the assessment of ovarian reserve and the localization of primordial follicles.

According to IVF outcomes using transplanted mouse ovaries with OCT examination, we demonstrated a strong correlation between the number of extracted oocytes and the OCT findings. Consistent with earlier reports, OCT findings were well correlated with histological data^27^. Therefore, we concluded that OCT examination could potentially be used to estimate ovarian reserve and IVF outcome in a similar manner to current tests such as serum AMH and basal follicle stimulation hormone levels and antral follicle counts.

Concerning clinical application, however, OCT examination of a whole human ovary would require prolonged exposure to near-infrared light (NIR) for image acquisition. Another issue is that OCT examination is best suited for flat surfaces as opposed to the round shape of the ovary. In present study, the maximum depth of examination was 100 μm. Therefore, unfortunately, the current commercial OCT could not be used to count the number of developing follicles because they are at a depth of up to 3 mm in the human ovarian cortex^29^,^30^; in particular, early human follicles were detected within 0.75 mm from the surface of the ovarian cortices^31^. Although OCT could assess the ovarian reserve and localization of thin-sliced human ovarian cortex with embedding and fixation^27^, these issues need resolution before clinical application will be possible to assess entire ovarian reserve of human being.

Currently, ovarian tissue cryopreservation is one of the key strategies in fertility preservation for young cancer patients who face imminent chemotherapy or radiation therapy, such as cyclophosphamide chemotherapy, which have deleterious effect for ovarian reserve^32^. Although ovarian tissue cryopreservation is still considered to be experimental, it is the only option in girls because there is no standard modality available for fertility preservation in girls^33^,^34^. Including our cases, there have been over 60 live births from transplanted ovarian tissues reported^8^,^35^. Recently, the first successful case of fertility restoration from ovarian tissue cryopreserved before menarche was reported^36^. Although accurate information regarding post-transplantation pregnancy rate is still limited, the pregnancy rate for ovarian tissue transplant was reported to be 18.3 to 36.3%^32^,^34^,^35^. While ovarian tissue cryopreservation techniques are advancing, many challenges remain for optimizing them. Remaining challenges include the efficacy of transplanted ovarian tissues, which is related to the ovarian reserve of patient, the method of ovarian tissue preparation, freezing-thawing techniques, the amount of ovarian tissue available for transplantation, transplantation techniques, and graft sites^34^,^37^. The present study indicates that OCT may be able to resolve these concerns by clarifying the number and localization of follicles in ovarian tissue. It is possible for OCT to aid in selecting the best ovarian tissue for ovary tissue transplantation. With ovarian tissue transplantation, the greatest concern would be in reintroducing metastatic malignancy following transplantation. To solve this problem, the OCT technique may be able to screen for cancer metastases present in ovarian tissue prior to transplantation^27^. Also, OCT may advance basic research in the area of ovarian tissue cryopreservation because it allows researchers to accurately count follicles in the ovary before and after ovarian tissue transplantation. Therefore, OCT will promote the development of methodologies for ovarian tissue cryopreservation and transplantation.

There are no reports from standpoint of OCT safety in reproductive health; although, the viability of ovarian tissue has been examined using the glucose uptake assay and neutral red staining^27^. A previous study investigated the thermal effects of its zona drilling on bovine and murine embryos^38^, however, heat effects caused of near infrared light exposure is still unknown. Therefore, the use of NIR technology in assisted reproduction is currently being investigated^39^. Currently, NIR laser-assisted embryo hatching is a common method in the area of assisted reproductive technology that aims to increase implantation and pregnancy rates. Although the clinical efficacy of laser-assisted hatching remains controversial^40^,^41^, NIR has been used widely in reproductive medicine and researchers have seen no evidence that the embryos are damaged during the laser hatching procedure^42^. For IVF applications, lasers now use near infrared light allowing for successful applications and minimizing DNA damage to the blastomeres^38^. In addition, NIR spectroscopic analysis has been applied to oocyte viability assessment for non-invasive clinical research^39^. In our study, we demonstrated that successive OCT and IVF protocols were not a detriment to the numbers of extracted oocytes, fertilization rate, or the blastocyst rate compared with IVF without OCT examination. Also, OCT examination was not harmful the placenta or offspring weight. Although there was significant difference on the birth weight, there was no difference on placenta weight. It is a just speculation, increasing of birth weight was due to some individual difference. In addition, OCT examination did not increase birth defects in offspring, and the offspring from transplanted ovaries had normal reproductive capacity. Certainly, our study alone is not sufficient to prove the absolute safety of NIR exposure used in OCT examination, but present study is the first to report evidence for reproductive safety in the application of OCT examination. If OCT examination is to be used in clinical application, the safety of longer exposure NIR times must be investigated.

In conclusion, OCT is a new and likely safe tool for assessing ovarian reserve without the need for fixation. With optimization for use in human ovarian tissue, it may eventually be used to directly count the number of primordial and developing follicles, which are different from currently available indirect methods such as serum AMH level, basal follicle stimulation hormone level, and antral follicle count. Lastly, the ability to know the accurate number and localization of developing follicles via OCT will allow for its use in ovarian tissue transplantation procedures, and be very helpful for patients who receive ovarian tissue transplantation for future pregnancy.

## Materials and Methods

### Experimental animals

Day 3, 10, and 21, and 30 and 50 week old female ICR mice (CLEA Japan and Japan SLC, Tokyo, Japan) were housed under a 12 hours light/dark cycle at 22 °C and fed *ad libitum* for ovarian tissue harvest. Under these housing conditions, 6 weeks old female ICR mice (Japan SLC, Tokyo, Japan) acted as ovarian transplantation recipients, and 10 weeks old female ICR mice acted as host mothers (Japan SLC, Tokyo, Japan). The experimental protocols and animal-handling procedures were performed with the approval of the Institutional Animal Care and Use Committee (IACUC) of St. Marianna University School of Medicine. Dissection, ovarian tissue transplantation, and *in vitro* fertilization were performed according to IACUC approved protocols.

### Preparation of mouse ovaries

Mice ovaries were obtained from mice of various ages as outlined above. Harvested ovaries were stored for 8–9 hours prior to OCT examination and ovarian tissue transplantation on ice in DMEM culture medium (Gibco, Thermo Fisher Scientific, MA, USA) supplemented with 10% FBS (Foetal Bovine Serum; Bovogen Biologicals, Melbourne, Australia) and 1% of antibiotics (Anti-Anti; Gibco, Thermo Fisher Scientific, MA, USA).

### Optical coherence tomography examination

The latest OCT system (Light-CT scanner; LL Tech, Paris, France) was used in present study. The mechanical characteristic of Light-CT scanner included images with 1 μm resolution in all three dimensions and 3D views that can be manipulated using a standard DICOM viewer. The wavelength of the OCT examination was 700 nm, the width of the wave was 125 nm, and the area of examination was 800 μm^2^.

To compare standard histological with OCT images of the ovary, mouse ovaries from each age group (day 3, 10, 21 and 30 weeks and 50 weeks) were examined using the OCT system. And ovaries for transplantation to kidney (as stated below) were also examined by same system. Images were taken up to a depth of 100 μm which was maximum depth at which high resolution images can be taken. The average exposure time during the OCT examination was 4–5 min.

### Histological study of mouse ovary

Ovaries examined with OCT were fixed by Bouin’s solution (Polysciences inc., PA, USA) and embedded in paraffin wax for sectioning (5 μm thick) followed by standard hematoxylin-and-eosin staining for later comparison.

### Ovary tissue transplantation

To verify the accuracy of OCT ovarian reserve estimation, ovarian tissue transplantation and IVF were performed, thereafter we compared the relationship between OCT images and outcome of *in vitro* fertilization (IVF). According to our previous study^43^, ovarian tissue transplantation must be performed within 8 hours of ovary extraction to maintain a normal IVF outcome.

Paired ovaries (day 3, 30 weeks, or 50 weeks old) examined by OCT were inserted, one under each kidney capsule, into the same 6 weeks old host mouse that had been ovariectomized to increase endogenous gonadotropin levels. To conform ovarian tissue transplantation efficiency, ovaries of 30 weeks old mice were cut into half, and the 50 weeks old mice ovaries were cut into pieces of the same size as that of day3 mice ovaries. And also, ovaries of day3 mice without OCT examination were prepared and transplanted to serve as a control group.

### *In vitro* fertilization and embryo transfer, breeding

In addition, to confirm the safety of OCT examination, IVFs were performed after ovarian tissue transplantation. To retrieve the first wave of oocytes from the ovaries, host mice were treated with 5 IU Serotropin (ASKA Pharmaceutical Co., Ltd, Tokyo, Japan) injections two weeks after ovarian tissue transplantation, followed by an intraperitoneal injection of 10 IU human chorionic gonadotrophin (hCG, Gonatropin; ASKA Pharmaceutical Co., Ltd, Tokyo, Japan) 48 hours later. Cumulus oocyte complexes (COCs) were collected from the ovaries and fallopian tube ampulla 14 hours hCG injection to compare the number of retrieved oocytes between day 3 mouse ovaries versus 30 weeks or 50 weeks old mouse ovaries. Later, retrieved oocytes were inseminated and cultured to the blastocyst stage for implantation into 10 weeks old ICR host mice. After embryo transfer and resultant pregnancy, newborn mice were delivered by Cesarean section. These resulting progeny were bred to screen for anomalies resulting from OCT examination.

### Statistical analysis

The JMP Pro version 12 program (SAS Institute Inc., NC, USA) was used for statistical analysis. The extracted oocyte number from transplanted ovaries, number of fertilized oocytes and blastocysts, and the fertilization and blastocyst rates were expressed as the mean + standard error (SE). A student t-Test test was performed and a P value of *p* < 0.05 was considered significant.

## Additional Information

**How to cite this article**: Takae, S. *et al*. Accuracy and safety verification of ovarian reserve assessment technique for ovarian tissue transplantation using optical coherence tomography in mice ovary. *Sci. Rep.* **7**, 43550; doi: 10.1038/srep43550 (2017).

**Publisher's note:** Springer Nature remains neutral with regard to jurisdictional claims in published maps and institutional affiliations.
